# Emotional Experience and Regulation in Juvenile Primary Headaches: A Cross-Sectional Pilot Study

**DOI:** 10.3390/children9111630

**Published:** 2022-10-26

**Authors:** Marina Miscioscia, Daniela Di Riso, Silvia Spaggiari, Mikael Poli, Giacomo Gaiga, Giacomo Randazzo, Maria Federica Pelizza, Laura Galdiolo, Alessia Raffagnato, Stefano Sartori, Irene Toldo

**Affiliations:** 1 Department of Developmental Psychology and Socialization, University of Padova, 35121 Padova, Italy; 2 Neuropsychiatric Unit, Department of Women’s and Children’s Health, University Hospital of Padua, 35128 Padova, Italy; 3 Juvenile Headache Center, Pediatric Neurology and Neurophysiology Unit, Department of Woman’s and Child’s Health, University Hospital of Padua, 35128 Padova, Italy

**Keywords:** migraine, tension-type headache, children and adolescents, emotional experience and regulation

## Abstract

A headache is the most common neurological symptom in children. Its subtypes are migraine (MH) and tension-type headache (TTH). Internalizing rather than externalizing symptoms are more frequent in children with headaches, but little is known about the reasons why. We aim to: (a) examine the interplay between emotional experience, affective regulation, and internalizing symptoms in children suffering from primary headaches and their caregivers; (b) identify potential predictors of children with migraines’ internalizing symptoms. Fifty children and adolescents with a diagnosis of primary headaches and their caregivers were compared to a sample of fifty-one healthy peers and caregivers. Self-reports and parent-reports were administered. Results indicate higher negative affect and internalizing symptoms and lower bodily awareness of emotions in the clinical sample (*n* = 50; Mage = 11.66, *SD* = 2.25) compared to controls (*n* = 51; Mage = 11.73, *SD* = 2.32); mothers of TTH children self-reported lower emotional awareness and higher difficulties in engaging in goal-directed behavior; a higher frequency of headaches was associated with greater emotional regulation difficulties. Internalizing symptoms were predicted by higher self-reported negative affect and parent-reported internalizing symptoms, and lower self-reported ability in the verbal sharing of emotions. These findings suggest the importance of assessing the psychological features linked to children with primary headaches’ psychological well-being.

## 1. Introduction

A headache is the most common neurological symptom and the most frequent cause of pain in children; its prevalence increases from childhood to adolescence [1,2], from 47.2% in 7- to 9-year-olds to 69.5% at the ages of 13–15 [2]. A headache is also a common neurological symptom of other medical conditions [3,4].

The most common headache subtypes are migraine (MH) and tension-type headache (TTH) [2]; their prevalence in pediatric populations is, respectively, 9.1% and 57.5% [2]. Their diagnostic criteria are reported in the International Classification of Headache Disorders [5].

Headaches can be a disabling disorder at any age, especially when characterized by intense and frequent attacks [6,7]. It causes school absenteeism, compromises school and out-of-school activities [6,8], provokes sleep disorders [4,6,7,8], and negatively influences educational skills [7,8,9,10,11].

The psychological symptoms of children with headaches have been widely explored: the literature mainly reports a higher prevalence of somatic complaints and higher levels of internalizing symptoms, depression, anxiety, and social problems such as difficulties in relationship engagement and infantilized behaviors in children with MHs compared to control individuals [6,12,13,14]. Moreover, among children and adolescents with emotional problems or in young patients with depression and anxiety, a headache is the most frequent physical symptom [12]. Weaker evidence suggests that children with MHs present externalizing symptoms such as rule-breaking, aggressiveness, and hyperactivity more often than their healthy peers [6,15,16]. Some authors suggest the existence of a typical personality for youths with migraines, as these children tend to be more sensitive, rigid, and self-controlling, show emotional rigidity, and repress anger and aggression, usually exhibiting worse overall adjustment [17].

Less is known about the reasons for the greater presence of internalizing symptoms in children with primary headaches. When it comes to this topic, emotional regulation can be considered one of the possible factors involved. Some authors hypothesize that emotional regulation mediates the association between somatic symptoms in general and internalizing symptoms [18]. It can be supposed that an inability to or avoiding identifying and expressing emotions may result in somatic symptoms [6,19,20,21]. In 8- to 16-year-olds with migraines, Tarantino et al. [22] found that a higher attack frequency was linked to a higher tendency to inhibit anger expression.

When it comes to family, Cerutti et al. [19] noted that if parents experience difficulties in emotional expression and regulation, they may not sustain their children in developing emotional self-regulation, thus reinforcing the tendency to somatization. Indeed, mothers of children with MHs seem to have greater difficulties in identifying feelings than mothers of healthy controls. In fact, empirical evidence shows that the family system has an important role in pediatric headaches [15,23]. Firstly, there seems to be a higher prevalence of headache familial recurrence [12]. Moreover, children with MHs are reported to have greater family functioning difficulties compared to healthy peers; their parents tend to experience higher levels of anxiety and mood disorders [6]. A study reports that mothers of children with MHs present significantly higher somatic symptoms [24]. As the literature mentions, parents of youths with migraines are likely to experience higher levels of parenting stress, to be overprotective and oversensitive towards issues concerning their children’s health, and to not offer them proper affective responsiveness [24,25,26].

Another topic that still needs to be studied more deeply regards the specificity of each headache subtype. Most studies focus on the MH vs. TTH [6], showing controversial results. Some authors report no significant psychological differences between children and adolescents of the two groups [10]. Others show that children with MHs experience greater difficulties in somatic, internalizing, anxiety–depressive, conduct, hyperactivity–inattention, and social domains [6,13,17]. Conversely, other studies report that pediatric patients with TTHs have greater anxiety and depression symptoms [27] and greater difficulty in identifying feelings than same-age MH sufferers [13,20]. Moreover, the attack frequency needs to be considered to identify different subtypes of pediatric headaches [22]. Very few studies about the psychological profiles of children with migraines with distinct headache attack frequency patterns and their parents are available. Some authors found that the attack frequency significantly impacts the psychosocial adjustment of children with headaches, leading to greater behavioral and emotional symptoms [13,17].

Considering all these aspects, the present work aims to examine the interplay between emotional experience, affective regulation, and internalizing symptoms in children suffering from different subtypes of primary headaches and their mothers through a cross-sectional case-control study design. We expect to find lower rates of emotional awareness and a higher presence of internalizing symptoms in children with primary headaches compared to healthy peers [6,17,18]. The same results are hypothesized for mothers of the clinical group compared to controls, based on the previously mentioned literature.

Moreover, this study aims to analyze the differences between children with MHs and TTHs and their mothers in their psychological functions in order to contribute to the understanding of the controversial results. Furthermore, the same psychological constructs will be assessed after creating subgroups of children with different frequencies of headache patterns and their mothers, as literature on this topic is scarce. We hypothesize that we will find worse outcomes in children with more frequent attacks and their mothers [13,17].

As a final step, this study aims to identify possible predictors of children with migraines’ internalizing symptoms; we assume the severity of headaches [20] and children’s and mothers’ emotional experience and emotional awareness [6,17,18,28] as the variables associated with the well-being of children with primary headaches.

## 2. Materials and Methods

### 2.1. Procedures

The present study was a cross-sectional case-control study involving pediatric patients followed at the Juvenile Headache Center in the Department of Women’s and Child’s Health of Padua University Hospital and their caregivers.

In recruiting participants, the following inclusion criteria were applied: age between 8 and 16 years old; a confirmed diagnosis of primary headache (MH or TTH) according to the diagnostic criteria of the International Classification of Headache Disorders (ICHD-3) [5]; a stable pattern of headaches in the last 6 months; and compliance to the clinical interview. The following were considered as exclusion criteria: age under 8 or over 16 years old; the presence of other chronic systemic neurologic or psychiatric diseases; poor comprehension of the Italian written language; and poor compliance to the clinical interview.

Participants were divided into subgroups based on medical conditions (i.e., symptom frequency and headache type). The first categorization was between patients whose symptom frequency included more than 4 episodes a month and those experiencing less than 4 episodes a month. This cut-off was chosen according to the literature that considers more than 4 attacks a month as an indicator for preventive treatment, representing a more severe condition that impacts quality of life [29].

A second subdivision concerned headache type: MH vs. TTH.

At the end of the outpatient control visit, patients and their caregivers were asked to participate in an adjunctive clinical interview based on self-report questionnaires focusing on psychological issues. Caregivers were informed that this adjunctive interview had clinical and research purposes. A detailed informed consent needed to be signed to join the survey. All caregivers gave their written consent for their and their children’s participation. Data were collected in the second semester of 2019.

During the control visit, the following clinical data were collected: gender, age at clinical visit, age at headache onset, type of headache, headache symptoms, headache diagnosis, time since diagnosis, frequency of headache attacks in the last 6 months, symptomatic drugs for headache (type and monthly frequency), and preventive pharmacological and non-pharmacological headache treatments.

After collecting informed consent, data about leisure activities such as the time a day spent using digital devices and sports were also collected.

Moreover, both parents and children completed self-report questionnaires (following the researcher’s instructions) in separate rooms at the clinic. The questionnaires for parents and children assessed emotional awareness (Emotion Awareness Questionnaire), emotional experience (Positive and Negative Affect Schedule), and psychological adjustment (Strength and Difficulties Questionnaire).

Data collection did not interfere with medical procedures. The compilation took about 45 min, both for the caregiver and the child.

The study protocol is part of a main project approved by the Institutional Review Board of Padua Hospital (Comitato Etico per la Sperimentazione Clinica della Provincia di Padova; protocol number 57897, approved on 20 October 2016). The research was performed in accordance with the Ethical and Deontological codes of Italian Psychologists. No reward was offered for participation.

As to the control sample, healthy children matched for age and sex to the clinical sample were recruited through convenience sampling. Their caregivers also participated in the study. As exclusion criteria, we considered the presence of primary headaches or other chronic, neurological, and psychiatric diseases and poor comprehension of the Italian written language. The control sample’s information was collected by trainees of the Department of Developmental and Socialization Psychology (University of Padova) under the full supervision of senior researchers. They contacted parents and, after obtaining their signed consent to participate for themselves and their children, arranged an appointment for the in-person compilation. The demographic information and the questionnaires were the same as for the clinical sample, except for the questions related to primary headaches.

### 2.2. Measures

#### 2.2.1. Children’s Psychological Functioning

The Strength and Difficulties Questionnaire (SDQ) [30] is a 25-item self-report behavioral screening tool assessing children’s psychological adjustment. It consists of 5 subscales: emotional symptoms, conduct problems, hyperactivity–inattention, peer relationships problems, prosocial behaviors, and a total difficulty score scale, obtained by adding the first five. In addition, internalizing and externalizing symptoms scales can be used. Higher scores indicate a more problematic psychological adjustment. Items are rated on a 3-point Likert scale, from 0 “not true” to 2 “certainly true”. The Italian version has been validated for 8- to 15-year-old children and adolescents [31]. The questionnaire demonstrated good validity and reliability [31]. In the present study, Cronbach’s alpha values for the total score (TDS), the internalizing symptoms scale (INT), the externalizing symptoms scale (EXT), the emotional symptoms (EMO), conduct problems (CON), hyperactivity–inattention (HYP), peer relationships problems (PEER), and prosocial behaviors (PROS) scales were α_TDS_ = 0.82, α_INT_ = 0.75, α_EXT_ = 0.71, α_EMO_ = 0.78, α_CON_ = 0.60, α_HYP_ = 0.58, α_PEER_ = 0.56, and α_PROS_ = 0.49.

The Emotion Awareness Questionnaire (EAQ) [32] is a self-report questionnaire assessing emotional awareness. Thirty items are rated on a 3-point Likert scale from 1 “not true” to 3 “often true”. Six scales represent 6 key aspects of emotion awareness: Differentiating Emotions (DE), Verbal Sharing of Emotions (VSE), Not Hiding Emotions (NHE), Bodily Awareness of Emotions (BAE), Attending to Others’ Emotions (ATOE), and Analyses of (Own) Emotions (AOOE). Higher scores indicate a higher presence of the ability, except for bodily awareness, in which higher scores correspond to lower attention to bodily symptoms of emotions. The Italian version demonstrated good validity and reliability [33]. In the present study, Cronbach’s alpha values for the 6 subscales were, respectively, α_DE_ = 0.81, α_VSE_ = 0.70, α_NHE_ = 0.76, α_BAE_ = 0.70, α_ATOE_ = 0.70, and α_AOOE_ = 0.72.

The Positive and Negative Affects Schedule (PANAS) [34] for Children is a 30-item self-report questionnaire assessing children’s emotional experience and, in particular, positive and negative affect. Items describe 15 positive words (e.g., happy) and 15 negative ones (e.g., sad) and are rated on a 5-point Likert scale from 1 “very little” to 5 “a lot”. A positive affect and a negative affect scale are calculated. Higher scores indicate a higher frequency of positive or negative affect experienced. Although the Italian version of the PANAS for adults has been validated [35], no Italian version is available for children, so we used a back-translation of the original version. In the present study, Cronbach’s alpha values for the Positive Affect Scale (POS) and the Negative Affect Scale (NEG) were α_POS_ = 0.86, α_NEG_ = 0.60, and α_EXT_ = 0.92.

#### 2.2.2. Parent’s Psychological Functioning

The Strength and Difficulties Questionnaire (SDQ) [36], parent version, is a 25-item questionnaire assessing children’s psychological adjustment from the parents’ point of view. The factors and the Likert scale are the same as the child version. Higher scores indicate a child’s more problematic adjustment. The Italian parent version has been validated and demonstrated good validity and reliability [37]. In the present study, Cronbach’s alpha values for the total score (TDS), the internalizing symptoms scale (INT), the externalizing symptoms scale (EXT), the emotional symptoms (EMO), conduct problems (CON), hyperactivity–inattention (HYP), peer relationships problems (PEER), and prosocial behaviors (PROS) scales were α_TDS_ = 0.79, α_INT_ = 0.71, α_EXT_ = 0.73, α_EMO_ = 0.63, α_CON_ = 0.52, α_HYP_ = 0.70, α_PEER_ = 0.66, and α_PROS_ = 0.66.

The Positive and Negative Affects Schedule (PANAS) [38] is the self-report version for adults of the PANAS-C. It has 30 items, 15 describing positive and 15 describing negative emotions, rated on a 5-point Likert scale from 1 “very little” to 5 “a lot”. As in the children’s version, a positive and a negative affect scale are calculated. The Italian version has good psychometric properties [35]. In the present study, Cronbach’s alpha values for the Positive Affect Scale (POS) and the Negative Affect Scale (NEG) were α_POS_ = 0.71 and α_NEG_ = 0.72.

The Difficulties in Emotion Regulation Scale (DERS) [39] is a 36-item questionnaire assessing clinically significant difficulties in emotion regulation. Participants respond on a 5-point Likert scale ranging from 1 “rarely” to 5 “almost always”. Six scales are computed: Nonacceptance of Emotional Responses (Nonacceptance), Difficulties Engaging in Goal-Directed Behavior (Goals), Impulse Control Difficulties (Impulse), Lack of Emotional Awareness (Awareness), Limited Access to Emotion Regulation Strategies (Strategies), and Lack of Emotional Clarity (Clarity). Higher scores indicate greater difficulties in emotion regulation. The Italian version demonstrated good validity and reliability [40]. In the present study, Cronbach’s alpha values for the 6 scales were α_nonacceptance_ = 0.84, α_goals_ = 0.71, α_impulse_ = 0.84, α_awareness_ = 0.71, α_strategies_ = 0.84, and α_clarity_ = 0.71.

### 2.3. Statistical Analysis

We performed three two-tailed Mann–Whitney *U* tests in order to check for differences between the clinical (1) and the control (2) groups (*n*_1_ min = 43, *n*_1_ max = 50; *n*_2_ min = 50, *n*_2_ max = 51), and, among the clinical sample, between 1) different diagnostic groups (MH vs. TTH) (*n*_MH_ min = 29, *n*_MH_ max = 31; *n*_TTH_ min = 14, *n*_TTH_ max = 19) and 2) frequency of headaches: more than 4 episodes per month vs. not (*n*_YES_ min = 17, *n*_YES_ max = 21; *n*_NO_ min = 24, *n*_NO_ max = 25). The rationale behind this categorization can be found in the Procedures Section. The comparisons were adjusted for potential confounders: we checked the presence of statistically significant differences in age and sex between all the groups using, respectively, Student’s *t*- and chi-squared tests. Moreover, we used Mann–Whitney *U* tests to assess the presence of significant differences between the clinical subgroups as to the number of hospitalizations. The variables we included in all three analyses were the child’s EAQ, PANAS, and SDQ scores, and the mother’s PANAS, SDQ, and DERS scores. For a significance level of 5%, a power analysis indicated a 70% chance of detecting a large effect size (*d* = 0.5) and a 32% chance of detecting a medium effect size (*d* = 0.3) as significant for a two-tailed test between two groups of *n*_1_ = 50 and *n*_2_ = 51.

We then computed a multiple linear regression model including the child’s PANAS–NEG and EAQ–VSE scores and the mother’s SDQ–INT and DERS–Awareness scores, in order to identify predictors of the children’s self-reported internalizing symptoms (the child’s SDQ–INT scale, our DV (dependent variable)) for the clinical group based on our hypotheses. For control purposes, the model also included the child’s number of hospitalizations, sex, and age variables. In building our model, we explored the correlation between the children’s self-reported internalizing symptoms (child’s SDQ–INT scale) and the proposed predictor variables using Pearson’s *r* coefficient.

A visual check of the assumptions of linearity (scatterplot with fitted density line) and homoscedasticity (residual vs. fitted values plot) indicated approximately linear relationships between the DV and the predictor variables and homogeneity of variances (see Supplementary Materials). Homoscedasticity was further confirmed via a Breusch–Pagan test (*χ*^2^ = 13.12; *df* = 7, *p* = 0.069). A Shapiro–Wilk test of normality indicated the normality of the distribution of the model’s residuals (*W* = 0.980; *p* = 0.635). The VIF (variance inflation factor) for our model was lower than the reference value of 2.60 (calculated through the following formula: 1/1-*R*^2^) for all predictor variables (sex = 1.22; age = 1.64; number of hospitalizations = 1.16; PANAS–NEG (child) = 1.50; EAQ–VSE = 1.75; SDQ–INT (mother) = 1.22; DERS–Awareness (mother) = 1.08), indicating no multicollinearity issues. Durbin–Watson test indicated no autocorrelation between the model’s residuals (*d* = 2.31; *p* = 0.346). A Shapiro–Wilk test of normality indicated that the dependent variable (SDQ-INT (child)) could be considered to approximate a normal distribution (*W* = 0.956; *p* = 0.060; *skewness* = 0.29; *kurtosis* = 2.03).

Finally, following a stepwise approach, we computed a second multiple linear regression model only including the variables that appeared to be statistically significant predictors of the children’s internalizing symptoms in the first model and compared the two using the *R*^2^ and *adjusted R*^2^ (*adj. R*^2^) coefficients of determination in order to find a better fit for our data.

Missing data were removed before proceeding with the regression analyses: for both linear regression models, *n* = 43. A power analysis indicated that a sample of 43 individuals was sufficient in model 2 for detecting as significant a moderate (*f*^2^ = 0.3) and a large (*f*^2^ = 0.5) effect size with an 82% and a 97% chance, respectively, at a significance level of 5%.

Data were analyzed using the statistical software R 4.1.2 [41] and the integrated development environment RStudio 2021.09.0 [42].

## 3. Results

Fifty children and adolescents with primary headaches, aged 8 to 16 years old (32 females and 18 males) were recruited for the present study among those attending the juvenile Headache Center in the Department of Women’s and Child’s Health of the Padua University Hospital in 2019. As for the clinical group, the diagnosis was made on average 2 years before the time of the study (*M* = 2.32, *SD* = 1.91); 74% of the children with migraines had never been hospitalized due to headaches, 20% had been hospitalized one time, and 6% more than one time; 67.3% of them had relatives with recurrent headaches; 98% used symptomatic drugs for headaches, 4% of them used analgesics more than 15 times a month for 3 months, 28% used preventive drugs for headaches, and 4% of them used non-pharmacological preventive treatments.

Almost 70% of those in line with the inclusion and exclusion criteria agreed to participate in the clinical interview. Reasons for refusal were time limits and lack of interest in the research. The final study sample included 50 participants (32 females).

As to the first categorization, 21 individuals had more than four episodes a month and 25 had less than four episodes a month.

As to the second subdivision, the MH and TTH groups were, respectively, of 31 and 19 participants. Moreover, 50 caregivers (45 mothers and 5 fathers) were included in the present research. Mothers of the clinical group were mainly clerks and office workers (30%) and had non-qualified professions, such as clerical and executive support in office activities, production, education, and health services (26%). Descriptive data are reported in Table 1.

As to the control sample, 51 healthy children aged 8 to 16 matched for age and sex to the clinical sample, as confirmed via the Student’s *t*- and chi-squared tests (*t* = −0.144, *p* = 0.886; *χ*^2^ *=* 0.005, *p* = 0.941), were recruited. Their caregivers also participated in the study; more specifically, 50 mothers and 1 father. Due to the fathers’ low response rate, we only considered data collected from the mothers for our analyses. Clinical and control samples’ mothers that joined the survey did not significantly differ in age (*t* = −0.33, *p* = 0.736) or professions (*U* = 1214.5, *p* = 0.761). Descriptive and demographic data are summarized in Table 1.

### 3.1. Group Comparisons

#### 3.1.1. Clinical vs. Control

The clinical and control groups were similar in age (*t* = −0.144, *p* = 0.886) and sex (*χ*^2^ = 0.005, *p* = 0.941). The clinical group presented higher levels of negative affect and children’s emotional and internalizing symptoms, as reported by both the child and the mother for all three variables. Furthermore, the clinical group also reported lower attention to bodily symptoms of emotions compared to controls. Results are reported in Table 2.

#### 3.1.2. MH vs. TTH

Children with MHs and TTHs were similar in age (*t* = −0.592, *p* = 0.556), sex (*χ*^2^ = 0.259, *p* = 0.610), and number of hospitalizations (*U* = 241, *p* = 0.167). Mothers of children suffering from MHs reported higher emotional awareness and lower difficulties in their own ability to engage in goal-directed behaviors compared to the mothers of children experiencing TTHs. Results are reported in Table 3.

#### 3.1.3. Frequency of Headaches > 4 per Month vs. <4

Children of the two groups were similar in age (*t* = −0.482, *p* = 0.632), sex (*χ*^2^ = 0.035, *p* = 0.850), and number of hospitalizations (*U* = 226, *p* = 0.294). Children in the group experiencing over four attacks per month reported hiding their emotions more frequently, while their mothers reported experiencing less emotional clarity, and reported their child as experiencing overall more adjustment difficulties. Results are reported in Table 4.

### 3.2. Predictors of Children’s Internalizing Symptoms for the Clinical Group

Correlations between the child’s self-reported severity of internalizing symptoms (SDQ–INT) and our proposed predictor variables are reported in Table 5. Table 6 includes model coefficients for our first model.

Higher levels of the child’s self-reported experience of negative affect (PANAS–NEG) and their experience of internalizing symptoms as reported by the mother (SDQ–INT) correspond to higher levels of the child’s self-reported internalizing symptoms; the inverse relationship can be observed for the EAQ–VSE scale, with lower levels of the child’s self-reported ability in sharing their emotions verbally being correlated with higher internalizing symptoms.

We computed a second multiple linear regression model because the correlation between the mothers’ DERS–Awareness scale and the DV was weak and not statistically significant and because our control variables (sex, age, and number of hospitalizations) showed no predictive value on our DV. Then, we compared the two models for a better fit. Model 2 included the child’s PANAS–NEG and EAQ–VSE scales, and the mother’s SDQ–internalizing symptoms scale as predictors. Model coefficients for model 2 and model comparisons between models 1 and 2 are reported in Table 6, as well as goodness-of-fit measures for both models.

Model 2 did not prove to be a better fit than model 1 in a statistically significant way, however, (a) the *adj.* *R*^2^ for model 1 was lower than its *R*^2^, indicating that the non-statistically significant variables were not providing any additional value; (b) the lower number of independent variables increased the error degrees of freedom (*df*), while the *adj. R*^2^ coefficient was virtually the same for the two models. Following these considerations, model 2 allows for more precise estimates. The model highlights a positive association between children’s self-reported severity of internalizing symptoms (SDQ–INT) and the child’s PANAS–NEG and the mother’s SDQ–NEG scores, and a negative association between the dependent variable and the child’s EAQ–VSE scores. Higher internalizing symptoms in children are predicted by higher levels of the child’s self-reported experience of negative affect, a higher rate of the child’s experience of internalizing symptoms as reported by the mother, and lower levels of the child’s self-reported ability in the verbal sharing of emotions. The model explains 57.7% of the variance in children’s self-reported internalizing symptoms score as measured through the SDQ (*R*^2^ = 0.557, *p* < 0.001).

## 4. Discussion

A headache is one of the most frequently reported somatic complaints in youth [1]. From a psychological perspective, the literature largely highlighted the higher presence of internalizing symptoms in pediatric patients suffering from this chronic disease [6,12,14], even though little research has focused on possible predictive factors of this pattern so far. Moreover, only a few studies have examined the emotional experience of children with different subtypes of headache (MH and TTH, and their subtypes, i.e., episodic and chronic MH, episodic and chronic TTH). The current cross-sectional pilot study aimed to assess the interplay between emotional experience, affective regulation, and internalizing symptoms in pediatric patients with primary headaches and their mothers, spreading light on the differences between the prevalent clinical headache subtypes.

According to the first hypothesis, our results are consistent with the literature reporting higher levels of negative affect and internalizing symptoms for children with headaches [6,12,14] compared to healthy controls. Curiously, children with headaches also reported lower attention to bodily symptoms of emotions. This result is probably due to the items’ formulation of the EAQ “Bodily Awareness of Emotions” scale (e.g., “when I am sad, I feel my body weaker”, “when I am feeling bad, I feel something different in my body too”). It may refer to the ability to connect feelings and somatic sensations, a lacking feature in children with migraines [6].

Mothers of the clinical group also noticed higher negative affect and internalizing problems in their children’s experience, compared to those of non-clinical youths. This aspect might be important as mothers seemed to be able to detect emotional fatigue in their children, despite the great somatic component of headaches.

According to the second hypothesis, comparisons between different headache subtypes were carried out. It might be useful to pay attention to these aspects, despite the small sample size and considering the explorative and descriptive nature of the present study. Literature about the comparison between children with MHs and TTHs is still in its infancy and has shown controversial results [6,12,13,27,43]. No differences emerged between MH and TTH children in relation to psychological functioning, emotional awareness, and positive and negative affect. Headache-related fatigue and sensory processing difficulties might equally affect the emotional functioning of these patients independently from the nature of the headache. The emotional experience of MH or TTH patients could be affected by the interplay of several contextual and psychological aspects, not explored in the present study.

As to mothers, those of children suffering from TTHs reported lower emotional awareness and higher difficulties in their own ability to engage in goal-directed behavior. As far as we know, no studies evaluated parental emotional regulation in mothers of children with headaches considering the distinction between MHs and TTHs. However, literature indicated that greater parental difficulties in emotional regulation may represent a risk factor for the onset of diseases with an important somatic component such as headaches [11,19], and this may also regard TTHs.

We propose some descriptive and explorative considerations also regarding the differences that emerged as to the frequency of headache patterns (>4/month vs. <4/month). Children experiencing four or more headache episodes per month, regardless of the diagnosis, reported more frequently hiding their emotions; a result which is consistent with our hypothesis and Tarantino et al.’s [28] study highlighting an association between a higher frequency of headaches and the tendency to inhibit anger expression. Our results showed that headache frequency and recurring experience of pain rather than subtype seems to affect or depend on the child’s emotional well-being.

Furthermore, mothers of children with a higher frequency of headaches reported experiencing less emotional clarity (DERS), and their child experiencing overall more adjustment difficulties, a result which appears once again consistent with our hypothesis and Cerutti et al.’s work [19]. A higher frequency of attacks may intuitively be linked to greater peer problems, as the literature suggested, via school absenteeism and social and activity limitations due to headaches [6,8,17].

According to our third hypothesis, the model highlighted a positive association between mothers’ reports of children’s internalizing symptoms and child reports of negative affect and internalizing symptoms. Moreover, a negative association between the children’s verbal sharing of emotions scale and the dependent variable was found. Higher internalizing symptoms in children were associated with higher self-reported negative affect, greater children’s internalizing symptoms as reported by mothers, and lower self-reported ability in the verbal sharing of emotions. Age, gender, and the number of hospitalizations did not appear to account for a significant part of children’s internalizing symptoms, but their emotional regulation did. Moreover, the less that children verbally shared their feelings, the more their internalizing symptoms increased. Youths with headaches are reported to experience difficulty in expressing their emotions [19]. Literature showed that the ability to identify, express, and regulate emotions plays a central role in influencing both children with migraines’ psychological well-being and somatic symptoms, as feelings can be processed and not somehow moved into the body [19]. Studies also highlighted that parents’ lower ability in emotional expression and regulation can negatively affect their children’s self-regulation of emotion and bodily experience, increasing their tendency to somatization [19,44].

As a pilot study, our results cannot be generalized and, given the cross-sectional nature of the work, no inference of causality can be drawn. Furthermore, the paper protocol was not pre-registered or pre-published. It is necessary to underline, as further limitations, the paucity of subjects in the clinical and control samples, mainly when it comes to the comparisons between groups with different patterns of headache frequency. Moreover, standardized questionnaires may fail in detecting the real-life experience of children, adolescents, and their mothers, and some of them are not validated on children’s populations. Missing data in the self- and parent-report questionnaires must be mentioned, in addition to the potential biases related to the inclusion of data only from mothers. Furthermore, youths with primary headaches in the present sample were selected among patients of a single medical clinic, instead of involving multiple hospitals, and they were all recruited in a tertiary child headache center rather than in a primary population. Lastly, being a pilot study with a limited number of subjects, medical treatment was not considered. The disproportionality in the sample size between subjects that use analgesics for acute attacks and subjects that use drugs or supplements for prophylaxis did not allow this in-depth exploration which needs attention in further studies.

The present work explored the emotional experience and regulation of youths with primary headaches and their parents by differentiating headache subgroups both as to the type (MH vs. TTH) and the frequency of attacks, contributing to filling this gap in the literature. The study confirmed the importance of assessing the psychological factors associated with well-being in children with primary headaches. As the literature assumes, the evaluation of children’s psychological characteristics using the SDQ can predict treatment outcomes and help provide appropriate initial interventions. Further studies should also involve fathers in order to assess potential differences with mothers, to better the knowledge about youths with migraines and their families’ psychological well-being, and the effectiveness of interventions. Moreover, parents’ and children’s self-esteem and self-efficacy may be relevant psychological correlates to explore for a deeper understanding of the process leading to internalizing symptoms.

## Figures and Tables

**Table 1:** Demographic information of parents and children. Means or percentages are reported.

**Children**		**Clinical Sample (*n* = 50)**		**Control Sample (*n* = 51)**	
		***M*** **/Percentage**	***SD***	***M*** **/Percentage**	***SD***
Age		11.66	2.25	11.73	2.32
Sex	Male	36% (*n* = 18)		35.3% (*n* = 18)	
	Female	64% (*n* = 32)		64.7% (*n* = 33)	
Time a day spent on video games, tablet, smartphone	1–2 h	66		58	
	3–4 h	23.4		28	
	>5 h	10.6		14	
Sports activities	Yes	78		90.2	
	No	22		9.8	
**Mothers**		**Clinical sample (*n* = 50)**		**Control sample (*n* = 51)**	
		***M*** **/Percentage**	***SD***	***M*** **/Percentage**	***SD***
Age		44.55	4.71	44.90	5.66

**Table 2:** Mann–Whitney *U* Test for the Clinical and Control Samples.

Variable		Group	*n*	Mean	*SD*	Mdn	*U*	*p*	r_rb_
Child	EAQ–Bodily awareness	Clinical	50	8.14	2.34	8.00	957	0.030	0.25
		Control	51	7.12	2.35	7.00			
	PANAS–Negative affect	Clinical	50	2.66	0.89	2.70	898	0.011	0.30
		Control	51	2.22	0.79	2.07			
	SDQ–Emotional symptoms	Clinical	50	4.56	2.55	4.00	946	0.024	0.26
		Control	51	3.37	2.48	3.00			
	SDQ–Internalizing symptoms	Clinical	50	5.88	3.36	5.00	962	0.033	0.25
		Control	51	4.49	3.36	4.00			
Mother	PANAS–Negative affect	Clinical	44	2.13	0.74	2.07	806	0.026	0.27
		Control	50	1.79	0.43	1.80			
	SDQ–Emotional symptoms	Clinical	43	3.42	2.04	3.00	535	<0.001	0.50
		Control	50	1.66	1.59	1.00			
	SDQ–Internalizing symptoms	Clinical	43	4.72	3.10	4.00	565	<0.001	0.47
		Control	50	2.3	2.04	2.00			

*Note*. r_rb_: rank biserial correlation.

**Table 3:** Mann–Whitney *U* Test for the MH and TTH Samples.

Variable		Group	*n*	Mean	*SD*	Mdn	*U*	*p*	r_rb_
Mother	DERS–Difficulties engaging in goal-directed behavior	MIG	30	11.33	3.18	11.50	141	0.041	0.38
		TTH	15	13.80	3.84	14.00			
	DERS–Lack of emotional awareness	MIG	30	13.53	3.52	13.00	141	0.043	0.37
		TTH	15	15.27	2.02	16.00			

*Note*. r_rb_: rank biserial correlation.

**Table 4:** Mann–Whitney *U* Test for the Different Frequency of Headache Samples.

Variable		Group	*n*	Mean	*SD*	Mdn	*U*	*p*	r_rb_
Child	EAQ–Not hiding emotions	No	25	10.56	2.90	11.00	170	0.040	0.35
		Yes	21	8.71	2.97	9.00			
Mother	DERS–Lack of emotional clarity	No	24	10.04	2.20	9.00	128	0.040	0.37
		Yes	17	10.94	1.52	11.00			
	SDQ–Total difficulties score	No	24	7.17	4.31	7.00	119	0.024	0.42
		Yes	17	11.82	6.78	14.00			

*Note:* “No” = Frequency of headache < 4 attacks/month; “Yes” = Frequency of headache > 4 attacks/month; r_rb_: rank biserial correlation.

**Table 5:** Correlation matrix between all variables included in model 1.

	SDQ–INT (Child)	Sex	Age	No. of Hospitalizations	PANAS–NEG (Child)	EAQ–VSE (Child)	SDQ–INT (Mother)	DERS–Awareness (Mother)
**SDQ–INT (child)**		0.15	−0.02	0.19	0.62 ***	−0.47 **	0.54 ***	0.07
**Sex**			−0.13	−0.15	0.24	−0.2	−0.04	0.12
**Age**				0.14	−0.17	−0.48 **	0.17	−0.05
**No. of hospitalizations**					0.21	−0.23	0.12	−0.06
**PANAS–NEG (child)**						−0.30 *	0.30	0.13
**EAQ–VSE (child)**							−0.20	−0.08
**SDQ–INT (mother)**								0.20
**DERS–Awareness (mother)**								

*Note*: *: *p* < 0.50; **: *p* < 0.05; ***: *p* < 0.001; PANAS–NEG = Positive and Negative Affect Scale–Negative Affect Scale; EAQ–VSE = Emotion Awareness Questionnaire–Verbal Sharing of Emotions scale; SDQ–INT = Strengths and Difficulties Questionnaire–Internalizing Symptoms scale; DERS–Awareness = Difficulties in Emotion Regulation Scale–Lack of Emotional Awareness scale.

**Table 6:** Model 1 and model 2 coefficients with the child’s SDQ–internalizing symptoms scale as the dependent variable, model fit measures for model 1 and model 2, and model comparisons.

**Model 1 coefficients**							
**Predictor**	**B**	**SE**	***t***	***p***			
Intercept	6.181	1.370	4.51	<0.001			
PANAS–NEG (child)	1.350	0.495	2.72	0.010			
EAQ–VSE (child)	−0.745	0.258	−2.89	0.006			
SDQ–INT (mother)	0.454	0.129	3.53	0.001			
Sex	−0.126	0.813	−0.15	0.878			
Age	−0.337	0.196	−1.72	0.095			
No. of hospitalizations	0.004	0.458	0.01	0.993			
DERS–Awareness (mother)	−0.101	0.115	−0.88	0.385			
**Model 2 coefficients**							
**Predictor**	**B**	**SE**	***t***	***p***			
Intercept	5.977	0.353	16.88	< 0.001			
PANAS–NEG (child)	1.657	0.436	3.80	< .001			
EAQ–VSE (child)	−0.745	0.258	−2.89	0.006			
SDQ–INT (mother)	0.394	0.122	3.22	0.003			
**Model fit measures**							
				**Overall model test**			
**Model**	**RSS**	***R*** **^2^**	***Adj. R*** **^2^**	***F***	***df*** **1**	***df*** **2**	***p***
1	190.89	0.616	0.539	8.02	7	35	< .001
2	210.04	0.577	0.545	17.76	3	39	< .001
**Model comparisons**							
**Model**	***SS***	***F***	***df***	***p***			
1–2	−19.147	0.87	−4	0.487			

*Note*. All independent variables with the exclusion of “sex” are mean-centered.

## Data Availability

Restrictions apply to the availability of these data. Data are available from the Principal Investigators (MM, DDR, and IT) upon reasonable request.

## Supplementary Materials

The following supporting information can be downloaded at: https://www.mdpi.com/article/10.3390/children9111630/s1, Figure S1: Scatterplot of the correlational matrix between all the variables included in the multiple linear regression model; Figure S2: Residual vs fitted values plot to verify the assumption of homoscedasticity in the multiple linear regression model.
