# Supplementary material for: Emotional Experience and Regulation in Juvenile Primary Headaches: A Cross-Sectional Pilot Study
Source: Children (Basel). 2022 Oct 26;9(11):1630. doi: 10.3390/children9111630 (PMC9688696; doi:10.3390/children9111630)
Supplement: Supplementary file 1 [file children-09-01630-s001.zip › children-1953294-supplementary.pdf]

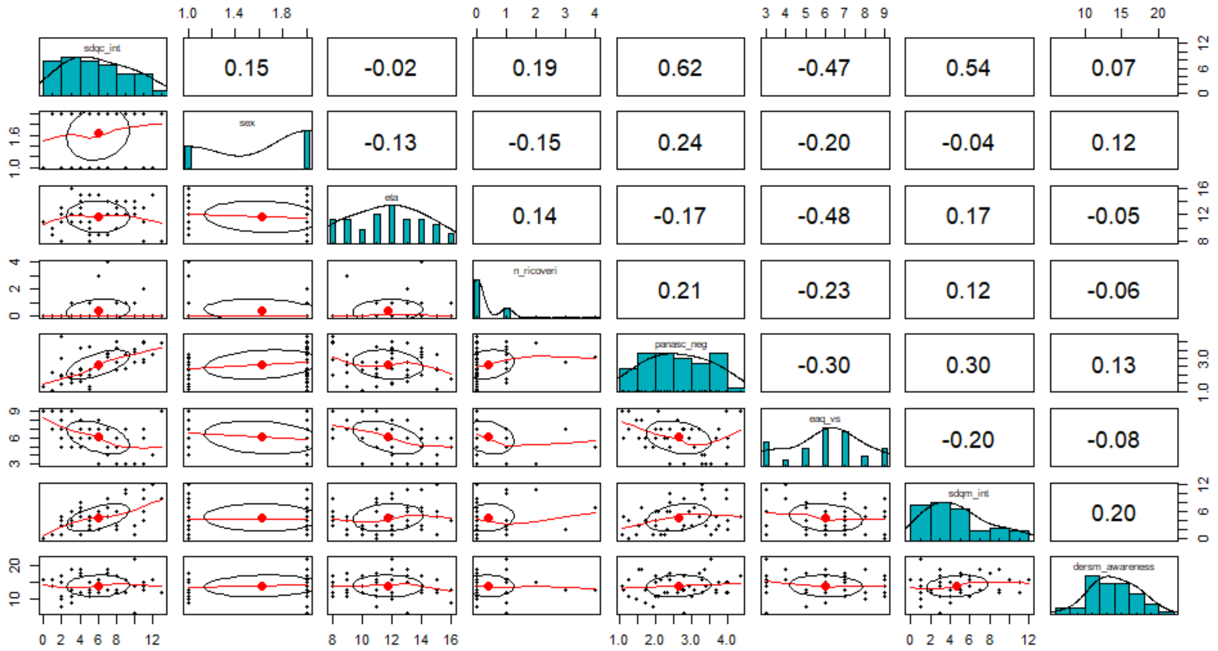

Figure S1. Scatterplot of the correlational matrix between all the variables included in the multiple linear regression model.

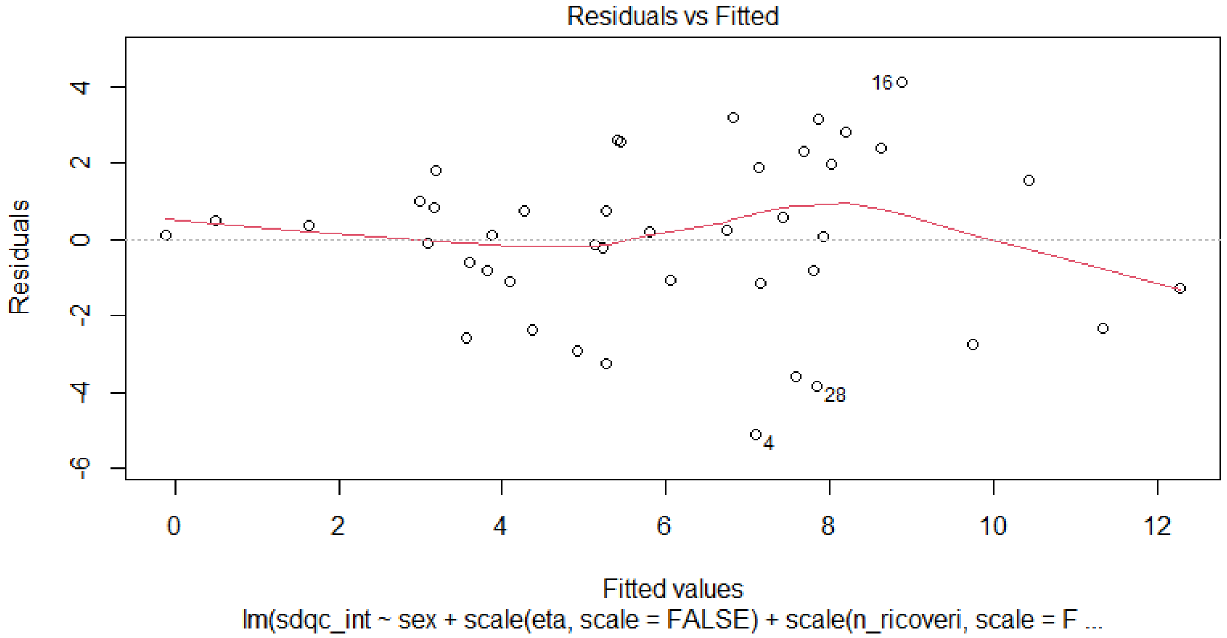

Figure S2. Residual vs fitted values plot to verify the assumption of homoscedasticity in the multiple linear regression model
